# Editorial: Heavy-Work Investment: Divergent perspectives

**DOI:** 10.3389/fpsyg.2023.1115928

**Published:** 2023-02-23

**Authors:** Aharon Tziner, Christian Vandenberghe, Julio César Acosta-Prado

**Affiliations:** ^1^Behavioral Sciences and Business Administration School, Peres Academic Center, Rehovot, Israel; ^2^HEC Montréal, Université de Montréal, Montreal, QC, Canada; ^3^Department of Business, University of the Pacific (Peru), Lima, Peru

**Keywords:** Heavy-Work Investment, involvement, human resource management, commitment, performance

Since the early 1970s, there have been concrete and robust testimonies to the centrality of work in people's lives, much beyond economic considerations (Diefendorff et al., 2002; Snir and Harpaz, 2012; Tziner et al., 2014, 2022). The experience of working is larger than the job itself, which explains why many of us devote most of their waking hours to work, beyond any other human activity. Recently, there has been a considerable increase in the time invested in work, partly as a by-product of the greater accessibility to technology and industrial competition (Tabak et al., 2021). Regardless of this trend, research has revealed individual differences in the devotion of time to work. One of the pioneering works that attempted to address those differences was Oates's (1971) research on workaholism. Since then, academically and in everyday use, workaholism has been the prominent expression of Heavy-Work Investment (van Beek et al., 2012).

Snir and Harpaz introduced the Heavy-Work Investment (HWI) concept in 2012. HWI encompasses working long hours and exerting much effort (physical and mental) at work. Snir and Harpaz (2015) proposed that HWI mediates between its different predictors (such as job engagement, addiction to work, financial needs, and employer demands) and individual outcomes (such as health and work satisfaction) with potential moderators (such as job type and fairness) (Snir and Harpaz, 2021).

This phenomenon of HWI is prevalent in nine countries around the world (Shkoler et al., 2021). However, HWI raises an interesting and important question: is HWI good or bad for the organization and its workers? Responses to this question are outlined below.

HWI can have a deleterious effect on workers. Long hours, in particular, appear to account for many negative consequences (Afota et al., 2021), including complaints associated with health and even illness, mistakes at work, occupational injuries, and workplace accidents, among a variety of additional debilitating factors described in the literature (Caruso, 2006; Snir and Harpaz, 2013). Notably, working overtime prolongs the duration of effort invested in the tasks, while the time for recovery from the exertion is shortened (Van Der Hulst and Geurts, 2001). Moreover, increased time spent on the job also means more extended exposure to workplace hazards, leading to less time to attend to non-work responsibilities (Caruso, 2006).

Despite these hazards, HWI can produce several positive outcomes. For example, Shamai et al. (2012) found that average “life satisfaction” was higher among employees working >50 h per week than among those working 36–50 h weekly. Apparently, the former group of employees experiences more “flow” than the latter group and, consequently, could be expected to report greater levels of positive affect (Shamai, 2015; Tabak et al., 2021).

The findings summarized above indicate that HWI may have both bright and dark sides. To disentangle the various (positive vs. negative) effects of and mechanisms associated with HWI, one needs to know more about the situational enhancers (e.g., work practices, task characteristics, leadership) and individual dispositions (e.g., sense of accountability, workaholism) that encourage employees' investment into their work role. Moreover, as prior research has identified both positive and negative implications for HWI, inquiry into what can drive these effects such as specific psychological processes and group dynamics as well as boundary conditions (e.g., individual values and personality, organizational practices fostering work-life balance) is warranted. The purpose of this Research Topic is to provide new knowledge about these issues, so as to offer a basis for future research avenues that can contribute to enhance our understanding of HWI's nature and implications.

Five papers are included in this topic. The first paper Shi and Cao offers a refreshing view of the role of proactive behavior in the workplace. The study indicates that high-commitment work systems (HCWSs) engender proactive behavior at work through the mediating influence of self-efficacy and career development prospects and that the tendency to conform to work rules strengthens the positive relationship between self-efficacy and employees' proactive behavior. These results fare well, particularly for employees wishing to build careers in their current places of employment, a seemingly rare phenomenon in today's changing world of work.

By contrast, the second paper Cheng et al. re-investigates the relationship between unreasonable tasks and work engagement from cognitive, affective, and resource-based perspectives—a valuable addition to established research. Based on the cognitive-affective systems theory and the job demands-resources model, the study constructs a chain mediation model in which unreasonable tasks negatively influence work engagement through work alienation and negative affect. The researchers further indicate how supervisor support can buffer the positive effect of unreasonable tasks on work alienation, thereby providing clearly valuable data for management practice.

The third paper Li et al. addresses the construct of self-accountability at work, i.e., the perceived expectation that one's actions will be evaluated and that compensation and penalties are predicated as such (Hall et al., 2006). Because self-accountability is a complex phenomenon with positive and negative outcomes, the research team highlighted the positive mediating role of obsessive passion [for work] between self-accountability and task performance and the positive mediating role of role overload between self-accountability and emotional exhaustion. The importance of the paper lies in its thought-provoking assessment of the benefits and drawbacks of felt accountability at work.

Based on the conservation of resources (COR) theory, the fourth investigation (conducted in China) Zeng and Liu offers insights into the positive relationship between workaholic leaders and employees' “defensive” self-presentation (e.g., faking work to impress supervisors)—achieved partly through the mediating mechanisms of employee workplace anxiety. Notably, segmentation (e.g., boundaries between work and leisure/family time) negatively moderated the relationship between workplace anxiety, self-presentation, and the overall mediating mechanism. The findings, albeit culturally based, offer directions for improving employee wellbeing and positive organizational outcomes.

The final offering in this collection Cheng and Gu employs a comprehensive ground-breaking meta-analysis that confirms that while workaholism, working excessively, and working compulsively, are positively associated with work performance, the relationships are stronger with contextual performance than with task performance. The subgroups analyses also indicate that the instrument used to measure workaholism moderates the relationships between workaholism and work performance, with the relationships being stronger when Robinson's (1999) Work Addiction Risk Test is being used. In contrast, the cultural variable of collectivism vs. individualism and the research design (cross-sectional vs. longitudinal) did not moderate the findings. These results can inform future work.

The five articles jointly contribute to enhance our understanding of the various facets of HWI and of the processes and boundary conditions associated with the construct. First, it appears that situational enhancers of employees' investment into their work are double-edged swords: they may engender positive outcomes (i.e., self-efficacy, proactive behavior, career development) when embedded within work practices that target high commitment, but they may have detrimental effects (e.g., alienation, negative affect, reduced job engagement) when they induce tasks that fall outside of employees' responsibilities. Second, supervisors may encourage unhealthy investment habits (ingratiation) among employees when they demonstrate workaholism to their teams. Third, employees themselves may have dispositions that make a difference: if they feel accountable at work, they may experience role overload and ultimately suffer from emotional exhaustion, while if they are workaholics (working excessively or compulsively) they may enjoy the benefit of higher extra-role performance. Finally, the current articles have identified situational factors that may buffer against the negative effects of work investment: supervisor support and an organizational climate that encourages segmenting the work role from one's personal life help reduce the negative effects of situational pressures to work harder. Overall, the articles presented in this Research Topic provide fine-tuned understanding of the facets of HWI, its enhancers, mechanisms, and moderators, and its various outcomes.

## Author contributions

All authors listed have made a substantial, direct, and intellectual contribution to the work and approved it for publication.
